# Diagnostic Ideas and Management Strategies for Thrombocytopenia of Unknown Causes in Pregnancy

**DOI:** 10.3389/fsurg.2022.799826

**Published:** 2022-04-06

**Authors:** Jie Li, Yue-Hua Gao, Jing Su, Lu Zhang, Yan Sun, Zeng-Yan Li

**Affiliations:** Department of Gynecology and Obstetrics, Tianjin Medical University General Hospital, Tianjin, China

**Keywords:** gestational thrombocytopenia, immune thrombocytopenia, neonatal alloimmune thrombocytopenia, prednisone, immunoglobulin, retrospective study

## Abstract

**Objective:**

To summarize the clinical characteristics and treatment options together with the maternal and neonatal prognoses in women with different degrees of thrombocytopenia of unknown causes during pregnancy.

**Materials and Methods:**

One hundred twenty-nine cases meeting the inclusion and exclusion criteria were retrospectively analyzed. Patients were divided into group A (50*10^9^/L) and group B (50*10^9^/L to 100*10^9^/L) according to the lowest level of platelet count during pregnancy. Patients were divided into those found to have thrombocytopenia in the relatively early, middle, and late stages according to the detection period of maternal thrombocytopenia during pregnancy.

**Results:**

There were 72 cases in group A, and 57 cases in group B. There existed statistically significant differences in terms of the proportion of primipara, the proportion with a history of thrombocytopenia, and the median length of pregnancy between the two groups (*p* < 0.05). The proportion of patients with severe thrombocytopenia as an indication for cesarean delivery was higher in group A than in group B (*p* < 0.05). More cases were detected at the relatively early stages of pregnancy in group A than in group B (*p* < 0.05). There was no difference in neonatal hemorrhage and events of thrombocytopenia between the two groups.

**Conclusion:**

Patients with platelet counts below 50*10^9^/L were mostly primipara with a history of thrombocytopenia, most often detected at a relatively early stage of pregnancy, and continued pregnancy might lead to aggravation of the disease. Combination therapy was required for patients with platelet counts below 30*10^9^/L to maintain the platelet counts within a safe range. Cesarean delivery was selected to terminate the pregnancies, and platelet counts should be raised above 50*10^9^/L before surgery. Close monitoring was required for those with platelet counts above 30*10^9^/L. There was no direct correlation between the maternal and neonatal platelet counts.

## Introduction

Thrombocytopenia in pregnancy is a common blood disorder during pregnancy, with a prevalence of approximately 5 to 10% (1). Thrombocytopenia in pregnancy has many causes and complex classifications, and different degrees of thrombocytopenia and the development trends due to different causes are closely correlated with the maternal and neonatal prognoses (2). In clinical practice, it is common to encounter thrombocytopenia of unknown causes in pregnancy, most of which are gestational thrombocytopenia (GT) and some of which are combined with primary immune thrombocytopenia (ITP). Neither pathogeneses are fully understood, but there is a presumed association with immune dysregulation during pregnancy. Clinical studies on this population are limited, with difficulty in differential diagnosis, unstandardized selection and application of platelet-raising drugs, and poor stratified management and treatment. In the present study, relevant clinical data were collected and analyzed, and the clinical characteristics and treatment options, together with maternal and neonatal prognoses, were summarized to share our experience to provide data support for the treatment and management of the population.

## Study Subjects and Methods

### Study Subjects

The clinical data of women with thrombocytopenia who delivered in the obstetric ward of the Tianjin Medical University General Hospital from 1 January 2018, to 31 May 2020, were retrospectively analyzed.

### The Inclusion and Exclusion Criteria

Singleton pregnancy cases with at least two platelet counts below 100*10^9^/L during pregnancy were included in the present study. Patients with PT with the following definite causes were excluded: ① those caused by gestational complications, such as those related to gestational hypertension and hemolysis, elevated liver enzymes, and low platelets (HELLP) syndrome. ② Those caused by systemic diseases during pregnancy, such as antiphospholipid syndrome (APS), systemic lupus erythematosus (SLE), and cardiopulmonary diseases. ③ Those caused by hematologic disorders such as leukemia, myelodysplastic syndrome, aplastic anemia, etc. ④ Others, including anticoagulation-induced pseudo-thrombocytopenia, multiple pregnancies, and patients with incomplete data.

### Data Collection

The maternal information including age, times of pregnancy/delivery, whether it was a primipara, previous history of thrombocytopenia, the stage when thrombocytopenia was first detected, platelet count at different stages, mode of delivery, surgical indication for cesarean section, the gestational week at delivery, coagulation indicators, glucocorticoids administration, application of gamma-globulin, blood product transfusion, amount of hemorrhage during delivery, postpartum hemorrhage, transferred to the intensive care unit (ICU), etc.

The neonatal information included live birth, birth weight and length, the presence of hemorrhagic events (including intracranial, gastrointestinal, and mucosal hemorrhages), platelet count within 24 hours after birth, whether the baby was transferred to the neonatal department, etc.

### Grouping and Stratification

The above cases were divided into group A (<50*10^9^/L) and group B (50*10^9^/L to 100*10^9^/L) according to the lowest level of platelet count during pregnancy. Patients in group A were further stratified into group I (<10*10^9^/L), group II (10*10^9^/L to 30*10^9^/L), and group III (30*10^9^/L to 50*10^9^/L). Four stages were included: pre-pregnancy, early, middle, and late stages, based on the different detection periods of maternal thrombocytopenia. Since most patients with thrombocytopenia first detected during early pregnancy actually had this condition before pregnancy, the data of those detected pre-pregnancy and during early pregnancy were also combined in the present study as the relatively early stage of pregnancy.

### Statistical Processing

The STATA 16.0 software was adopted for statistical analysis, and the GraphPad Prism 9.0 was used for charting. Medians combined with interquartile were used for statistical analysis of continuous variables, and the Student’s t-test for independent samples was used for comparison between two samples. Dichotomous variables were expressed as the number of cases combined with percentages, and the chi-square test or Fisher’s exact probability method were adopted for the comparison between two samples. *P* < 0.05 was considered statistically significant.

## Results

### Case Screening

**Figure 1:**
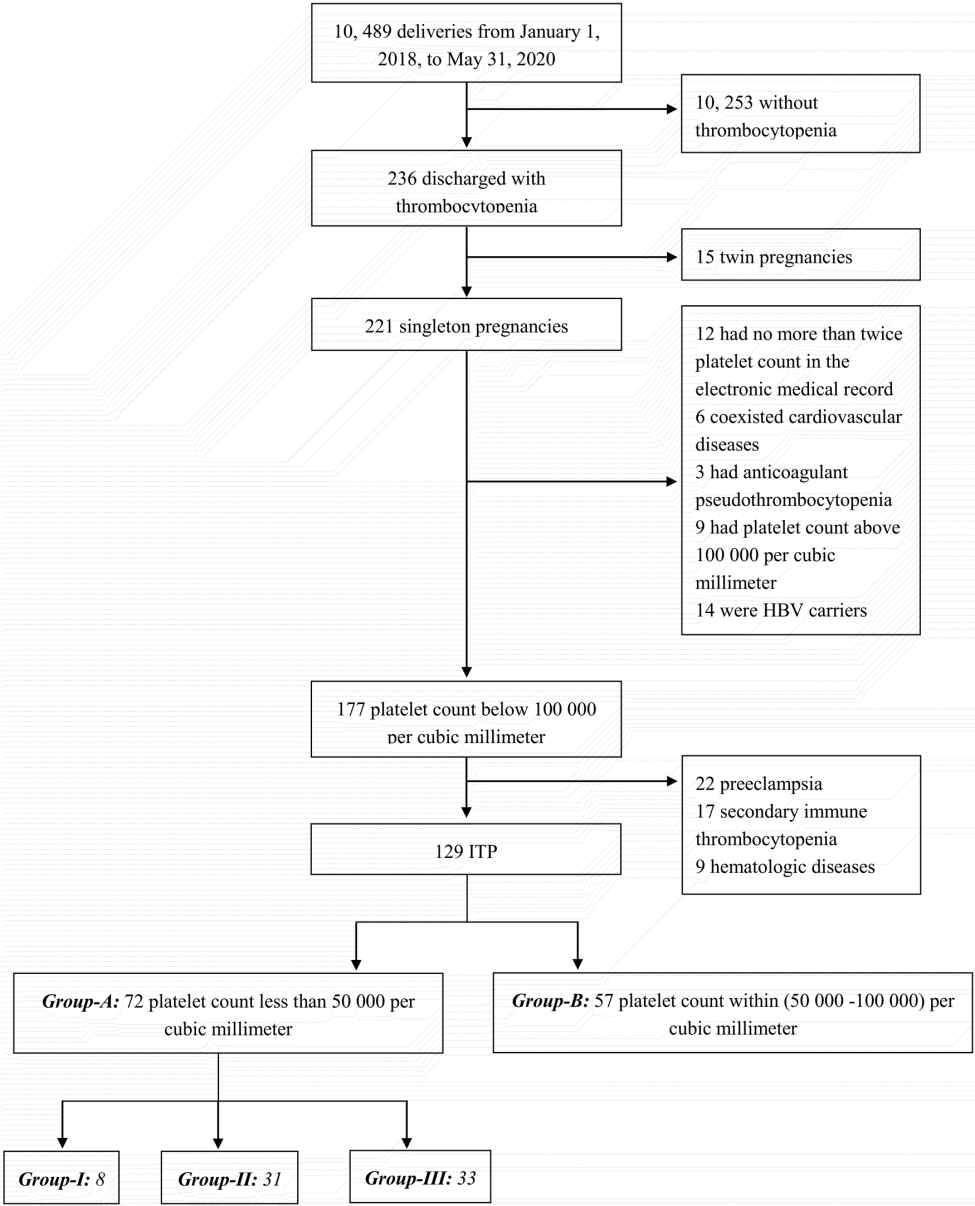
The case screening flow chart.

From 1 January 2018, to 31 May 2020, a total of 10,489 pregnant women delivered in the obstetric ward of the Tianjin Medical University General Hospital, of whom 236 had thrombocytopenia. After excluding thrombocytopenia due to a definite etiology (with the details referred to in the case screening flow chart in Figure 1), 129 cases were finally included in the present study. According to the grouping and stratification criteria, there were 72 cases in group A and 57 cases in group B. In group A, there were eight cases in group I, 31 in group II, and 33 in group III.

### Maternal Information

There was no statistically significant difference in the demographic characteristics between groups A and B (*p* > 0.05). Among the cases in group A, 73.6% (53/72) were primipara, 61.1% (44/72) had a history of thrombocytopenia, and the median gestational age was 267 days (38 ± 1 weeks). Among those in group B, 52.6% (30/57) were primipara, 24.6% (14/57) had a history of thrombocytopenia, and the median gestational age was 277 days (39 ± 4 weeks). There existed statistically significant differences in the above three indicators between the two groups (*p* = 0.01, *p* = 0.00, and *p* = 0.00, respectively). The cesarean delivery rates were 81.9% (59/72) and 70.2% (40/57) in groups A and B, respectively, with a slightly higher rate in group A than in group B, but the difference was not statistically significant (*p* = 0.12). The main indications for cesarean delivery were severe thrombocytopenia, scarred uterus, etc. In group A, 66.1% (39/59) of cases underwent cesarean delivery due to severe thrombocytopenia. In group B, only three out of 40 cases underwent cesarean delivery due to thrombocytopenia. The percentage of cesarean delivery indicated by “severe thrombocytopenia” was significantly higher in group A than in group B, and the difference was statistically significant (*p* = 0.00). Four (5.6%) cases had a postpartum hemorrhage in group A, and one (1.4%) was transferred to the ICU. While in group B, four (7.0%) cases had a postpartum hemorrhage, and three (5.3%) were transferred to the ICU. The differences in the above indicators were not statistically significant between the two groups (*p* > 0.05). The details are illustrated in Table 1.

**Table 1:** The maternal clinical characteristics.

	Group A (*n* = 72)	Group B (*n* = 57)	*p*
Age(y)	30 [28, 33]	31 [29,33]	0.45
Primipara	53 (73.6)	30 (52.6)	0.01
History of thrombocytopenia	44 (61.1)	14 (24.6)	0.00
Duration of pregnancy(d)	267 [261, 273]	277 [271, 281]	0.00
Cesarean delivery	59 (81.9)	40 (70.2)	0.12
Cesarean delivery due to thrombocytopenia	50 (84.7)	5 (12.5)	0.00
Hemorrhagic volume at delivery (ml)	300 [200, 400]	200 [200, 300]	0.96
Postpartum hemorrhage	4 (5.6)	4 (7.0)	0.73
Transferred to ICU	1 (1.4)	3 (5.3)	0.21
Length of hospital stay(d)	6 [5, 7]	5 [4, 7]	0.16

*Note: The countable data were expressed as n (%); The measurement data were expressed as M [IQR].*

### Neonatal Information

There was one case of fetal loss in groups A and B, with a gestational age of 18 ± 3 weeks and 27 ± 2 weeks, respectively. The neonatal live birth rates were 98.6% (71/72) in group A and 98.2% (56/57) in group B. In the statistics of data from all live births, it was found that there was a tendency for a statistically significant difference in birth weight (*p* = 0.055) and no statistically significant difference in birth length (*p* = 0.46) between the newborns in groups A and B.

**Figure 2:**
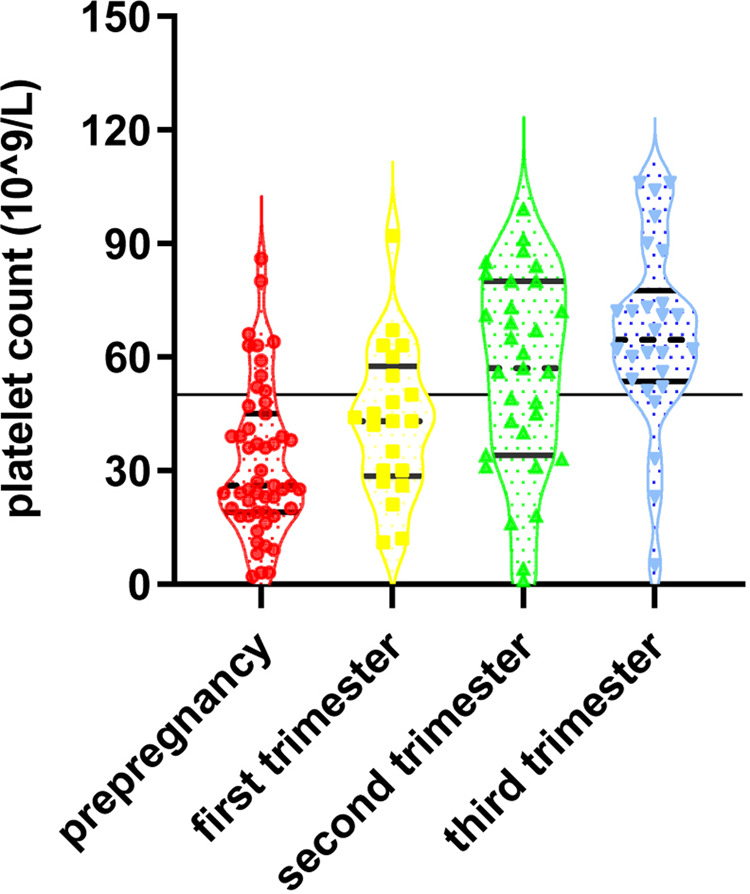
The platelet count in neonates of pregnant women with different degrees of thrombocytopenia.

Among the 39 live births transferred to the neonatal department, the median platelet count was 90*10^9^/L, of which 11 cases (eight in group A and three in group B) had a neonatal platelet count below 150*10^9^/L (28.2%), and 28 cases were within the normal range (71.8%). Twenty-six neonates (36.1%) in group A were referred to the neonatal department with a median platelet count of 209.5*10^9^/L. Three (4.2%) had hemorrhagic events, including one gastrointestinal hemorrhage, one intracranial hemorrhage, and one with gastrointestinal and intracranial hemorrhage. In the three cases with neonatal hemorrhagic events, they were not found to have reduced platelet counts, which were 222*10^9^/L, 254*10^9^/L, and 179*10^9^/L, respectively. In the three cases, the pregnant mothers already had reduced platelet counts before pregnancy, and the lowest values of platelets before delivery were 39*10^9^/L, 23*10^9^/L, and 22*10^9^/L, respectively. Thirteen newborns (29.7%) in group B were referred to the neonatal department with a median platelet count of 236*10^9^/L, and no neonatal hemorrhagic events were observed. There was no statistically significant difference in the number of cases transferred to the neonatal department, cases with neonatal thrombocytopenia, and the incidence of neonatal hemorrhagic events between groups A and B (p > 0.05). The details are demonstrated in Table 2, and the distribution of the platelet count in neonates is shown in Figure 2.

**Table 2:** The delivery information of neonates.

	Group A (*n* = 72)	Group B (*n* = 57)	*p*
Live birth	71 (98.6)	56 (98.2)	0.87
The hemorrhagic event^a^	3 (4.2)	0 (0.0)	0.12
Transferred to the neonate department^b^	26 (36.1)	13 (29.7)	0.16
The neonatal platelet count(10^9^ /L)^b^	210 [142, 265]	236 [168, 295]	0.81
Neonatal thrombocytopenia^b^	8 (30.8)	3 (23.1)	0.62
Weight at birth(g)^a^	3195 [2883, 3485]	3400 [3143, 3685]	0.06
Height at birth(cm)^b^	50 [48, 51]	51 [50, 52]	0.46

*Note: The countable data were expressed as n (%); The measurement data were expressed as M [IQR]; ^a^referred to the data in a live birth; ^b^referred to the data in neonates transferred to the neonate department.*

### Maternal Coagulation Function

All pregnant mothers in the present study were tested for coagulation at least once during hospitalization, and the tests included prothrombin time (PT), activated partial thromboplastin time (aPTT), fibrinogen (FI), thrombin time (TT), and D-dimers. No statistically significant difference was found between the two groups in the above coagulation indicators (*p* > 0.05). The details are shown in Table 3.

**Table 3:** The maternal coagulation function.

	Group A (*n* = 72)	Group B (*n* = 57)	*P*
The prothrombin time(s)	10.1 [9.8, 10.5]	10.2 [9.7, 10.5]	0.93
The activated partial prothrombin time(s)	25.6 [23.9, 27.4]	25.7 [24.9, 28.4]	0.28
Fibrinogen(g/L)	4.2 [3.45, 4.58]	4.4 [3.9, 4.7]	0.15
The thrombin time(s)	17.8 [16.7, 18.9]	17.6 [16.8, 18.5]	0.72
D-dimers(mg/L)	1672.0 [1052.5, 2591.8]	1632.0 [1134.3, 2342.5]	0.57

*Note: The countable data were expressed as n (%); The measurement data were expressed as M [IQR].*

### The Population Distribution of Different Degrees of Maternal Thrombocytopenia Found in Various Stages

Among the 129 cases, 51 cases with thrombocytopenia were detected before pregnancy, accounting for 39.53% of the total population. Twenty-one cases were detected in early pregnancy, accounting for 16.28% of the total population. Thirty-one cases were detected in middle pregnancy, accounting for 24.03%, and 26 cases were detected in late pregnancy, accounting for 20.16% of the total population. In group A, 76.39% (55/72) were identified to have thrombocytopenia in the early stages of pregnancy, 18.06% (13/72) in the middle of pregnancy, and only 5.56% (4/72) in the late stages of pregnancy. While in group B, 29.82% (17/57) were detected to have thrombocytopenia in the early stages of pregnancy, 31.58% (18/57) in the middle of pregnancy, and 38.60% (22/57) in the late stages of pregnancy. The proportion of cases in group A with reduced platelet counts at relatively early stages of pregnancy was significantly higher than in group B, and the difference was statistically significant (*p* = 0.00). While the proportion of cases in group B with reduced platelet counts detected at the late stages of pregnancy was significantly higher than in group A and the difference was statistically significant (*p* = 0.00). The details are illustrated in Table 4.

**Table 4:** The population distribution of pregnant women with different degrees of thrombocytopenia detected at different stages.

	Group A (*n* = 72)	Group B (*n* = 57)	*p*
Pre-pregnancy + the early stage	55 (76.39)	17 (29.82)	0.00
The middle pregnancy	13 (18.06)	18 (31.58)	0.07
The late pregnancy	4 (5.56)	22 (38.60)	0.00

*Note: The countable data were expressed as n (%).*

**Figure 3:**
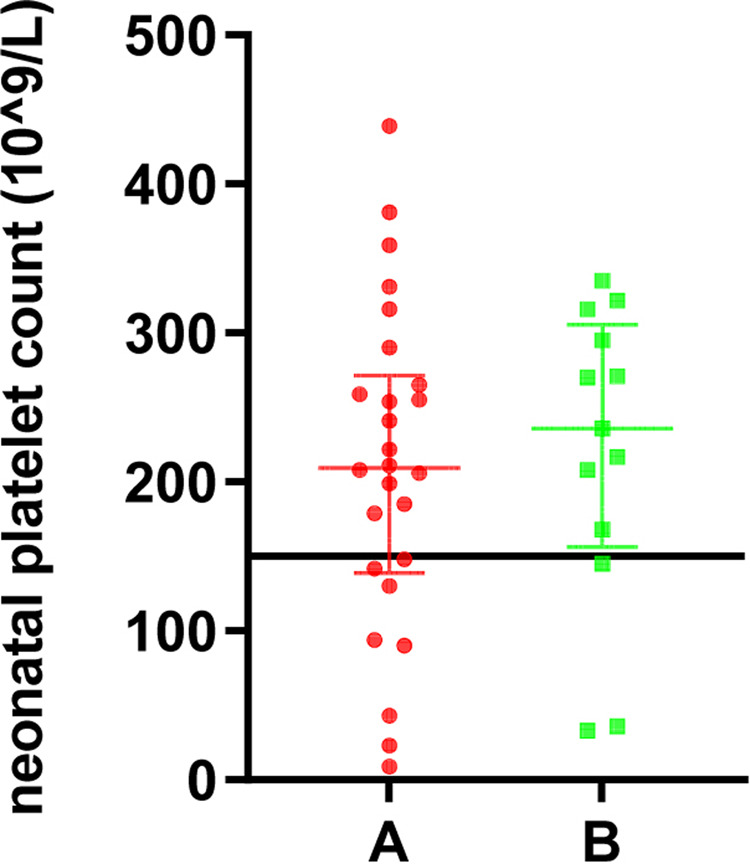
The distribution of platelet count in thrombocytopenia detected at different stages.

In the 51 cases with thrombocytopenia detected before pregnancy below the standard range of 50*10^9^/L, the distribution of each observation point was dense and uniform and the median platelet count was 26*10^9^/L. In the 21 cases diagnosed at an early stage, the median platelet count was 43*10^9^/L. In the 31 cases diagnosed at the middle stage, the median platelet count was 57*10^9^/L. While in 26 cases detected at a late stage below the standard line of 50*10^9^/L, the distribution of each observation point was dense and uniform and the median platelet count was 64.5*10^9^/L, as shown in Figure 3.

### Treatment Options

In most cases, combined platelet-raising therapy was conducted with platelet counts below 30*10^9^/L, i.e., groups I and II. There were eight cases in group I, accounting for 11.1% of cases in group A (8/72). All cases had received platelet-raising therapy with prednisone acetate combined with platelet transfusion. The proportion of cases with additional prednisone acetate combined with intravenous immune globulin(IVIG) and IVIG combined with platelet transfusion were 62.5% (5/8). There were 31 cases in group II, accounting for 43.1% (31/72) of the cases in group A, and 29.0% (24/31) of these patients received platelet transfusions, most of who needed to raise the platelet count above 50*10^9^/L before cesarean delivery, and 48.4% (15/31) received platelet-raising therapy with prednisone acetate combined with platelet transfusion. There were 33 cases in group III, accounting for 45.8% (33/72) of the cases in group A. Single platelet-raising therapy was conducted in most of the patients, with 24.2% (8/33) of patients in this group receiving preoperative platelet transfusions and another 9.1% (3/33) receiving prednisone acetate. In group B, 7.0% (4/57) received prednisone acetate, 5.3% (3/57) received IVIG, 5.3% (3/57) received platelet transfusions, and almost no case received combination therapy. The details are shown in Table 5.

**Table 5:** The selection of therapeutic protocol in pregnant women with different degrees of thrombocytopenia.

	Group A			Group B (*n* = 57)
	I (*n* = 8)	II (*n* = 31)	III (*n* = 33)	
A single administration of prednisone	0 (0.0)	0 (0.0)	3 (9.1)	4 (7.0)
A single application of IVIG	0 (0.0)	0 (0.0)	2 (6.1)	3 (5.3)
A single administration of platelet	0 (0.0)	9 (29.0)	8 (24.2)	3 (5.3)
Prednisone + IVIG	5 (62.5)	5 (16.1)	0 (0.0)	0(0.0)
Prednisone + platelet	8 (100.0)	15 (48.4)	1 (3.0)	1 (1.8)
IVIG+ platelet	5 (62.5)	5 (16.1)	1 (3.0)	0 (0.0)

*Note: The countable data were expressed as n (%).*

## Discussion

It was previously mentioned that the incidence of pregnancy thrombocytopenia pregnancy was approximately 5 to 10%. In the present study, the incidence of PT was 2.25% (236/10,489). This might be caused by the fact that the initial screening setting in the present study was for patients with platelet counts below 100*10^9^/L, and those with a platelet count in the range of 100*10^9^/L to 150*10^9^/L were not included. During normal pregnancy, the platelet count tends to decrease with the increase of gestational age but generally does not drop to 100*10^9^/L, and it can return to a normal level sooner after delivery. Therefore, it is usually considered to be clinically significant when the platelet count is below 100*10^9^/L (3). The most frequent and troubling cases in clinical practice are GT and ITP combined with pregnancy, and the differential diagnosis between the two is complicated. The platelet count in some patients with GT may return to normal after the termination of pregnancy or may develop ITP, and some patients with ITP may experience an exacerbation of the disease during pregnancy. The present study focused on the diagnosis and treatment of the two conditions.

### The Diagnosis and Differential Diagnosis of GT and ITP

It is mentioned in the newly published 2019 American College of Obstetricians and Gynecologists guidelines that both GT and ITP are considered exclusionary diagnoses. The specific diagnostic points for ITP in pregnancy can be referred to as the national expert consensus on ITP in adults. The differential diagnosis of GT and ITP could be achieved based on the following aspects: ① The diagnosis criteria were different. GT and ITP could be differentiated by platelet counts below 150*10^9^/L or 100*10^9^/L, respectively. Based on the combination of domestic and international guidelines, it was suggested that the diagnostic levels of platelet counts in ITP during pregnancy might be even lower. ② The onset stage was different: ITP in pregnancy tended to be present in early pregnancy or before pregnancy, while GT tended to occur in middle to late pregnancy or early after delivery. It has been suggested that the presence of a platelet count as low as 50*10^9^/L before 28 weeks of gestation might be a strong predictor for ITP and might be used as a reference to differentiate GT from ITP (4). ③ The clinical manifestations and treatment were different: platelet counts might drop further during pregnancy in patients with ITP, and without intervention, platelet nadir might occur in late pregnancy (5), with spontaneous hemorrhage of the skin, mucous membranes, and organs within a short period, and intracranial hemorrhage might occur in severe cases. Patients with GT, on the other hand, would not show obvious systemic discomfort. The physical examination might be primarily free of skin and mucosal hemorrhages, and the risk of significant organ hemorrhage could be extremely low, so the prognosis may be good as the platelet count could rapidly return to a normal range after delivery and generally would not require treatment. However, in clinical practice, the differential diagnoses of GT and ITP are still a major problem for clinicians.

### The Findings in the Present Study

Most of the cases with platelet counts below 50*10^9^/L occurred in the relatively early stage of pregnancy, with most of the cases being ITP in pregnancy. It has been shown (6) that approximately 80% of patients were first diagnosed with ITP in early pregnancy, and the median, minimum platelet count during pregnancy was 56*10^9^/L, which was similar to the percentage of 76.39% of patients in group A in the present study. In contrast, in patients who developed thrombocytopenia in the later stage of pregnancy, platelet counts were mostly in the range of 50*10^9^/L to 100*10^9^/L (*p* = 0.00). This was consistent with the latest edition of the ACOG practice bulletin on thrombocytopenia in pregnancy published in 2019, which stated that platelet counts in ITP patients in the early stage of pregnancy tended to be below 50*10^9^/L (2). In the present study, the proportion of cases with a history of thrombocytopenia was significantly higher in group A than in group B (*p* = 0.00). One study (7) suggested that approximately 64.8% of pregnant women with pre-existing ITP were not in remission during the current pregnancy, and approximately half had platelet counts as low as 50*10^9^/L. A case series reported in 2019 from the West China Second University Hospital (with 533 cases included) suggested that among cases with a history of thrombocytopenia, significantly more cases had platelet counts below 30*10^9^/L in the current pregnancy than those with platelet counts within the range of 30*10^9^/L to 50*10^9^/L (*p* = 0.00) (8). In the present study, the proportion of primipara was also significantly higher in group A than in group B (73.6% vs. 52.6%). The difference was statistically significant (*p* = 0.013), which differed from the previous study. This might be a new finding or a chance situation due to the limitation in sample size. Large samples and more representative study groups are needed in a future study to complement this point. In terms of pregnancy outcomes in both groups, the birth weight of newborns in group A was lower than in group B. There was a tendency for statistical differences between the two groups (*p* = 0.055), which could be due to the shorter length of pregnancy in group A than in group B cases (*p* = 0.00), the limitation in sample size, or the presence of immune disorders in the present population, which could affect fetal growth and development. More data is still needed in the future to draw more reliable conclusions.

Therefore, blood test screening in early pregnancy has clinical significance. For cases with a severe reduction in platelet counts, especially those with a history of thrombocytopenia, more intensive attention should be paid to increase laboratory tests such as platelet antibodies or even bone marrow aspiration as appropriate, to comprehensively assess the possible maternal and neonatal risks associated with continued pregnancy, and to advise a delay of pregnancy if necessary. The results of the present study also suggested that thrombocytopenia presenting in late pregnancy might be a more optimistic situation and should be monitored and observed during clinical management.

### Treatment Options

The goal of treatment is to raise the platelet count to a safe range rather than a normal range and avoid overtreatment. There is a lack of clear evidence-based medical data on whether patients with ITP should be treated before the start of pregnancy and at what level the platelet count should be maintained, while more emphasis was placed on the observational monitoring in patients with GT. The present study was also limited by the disadvantage of a retrospective study in that it was poorly documented whether patients received treatment before pregnancy and the specific treatment regimen. Currently, it is recommended by national and international experts that treatment should be based on platelet count and hemorrhagic tendency.

As with the first-line treatment for an adult with ITP, glucocorticoids and IVIG are the first treatment options for ITP in pregnancy. But it should be noted that the efficacy and safety of high doses of dexamethasone in pregnancy are not yet investigated. There might be teratogenic risks associated with the administration in early pregnancy. Thus, it is not recommended for application in pregnancy at present. Prednisone is recommended for ITP in pregnancy because it does not cross the placenta and is recommended for short cycles (three weeks) during middle and late pregnancy or before delivery, starting with a low dose of 10 mg/d up to 30 mg/d. In cases where oral glucocorticoids are ineffective, high-dose prednisone combined with IVIG or azathioprine is recommended. IVIG has the advantage of fast onset of action, with studies showing that IVIG takes effect 2 ± 1 days after injection compared to 16 ± 19 days for glucocorticoids (9). IVIG can be administered repeatedly during pregnancy or 3–5 days before the planned delivery at a recommended dose of 400 mg/kg.d^–1^ for 5–7 days. Combination therapy can also be used when a single treatment option fails to take effect. In the present study, the proportion of cases with combination therapy was significantly higher in group A than in group B. Those with platelet counts below 30*10^9^/L were mostly treated with combination therapy, while those with platelet counts above 30*10^9^/L did not need treatment, but were monitored and observed closely. As can be inferred from the summary in Table 5, the lower the platelet count, the higher the demand for various platelet-raising regimens, especially combination therapy. The most commonly used combination therapy was prednisone acetate combined with platelet regimens. The 2017 UK Guidelines for Platelet Transfusion states that the life cycle of platelets is 5–7 days and that platelet transfusions in patients with ITP should only be adopted as a means of resuscitation when other treatments are ineffective or when the condition is urgent because repeated platelet transfusions may induce the production of more platelet antibodies, thus aggravating the condition. In the present study, all cases below 10*10^9^/L (8/8) received platelet transfusions. Platelet transfusion was also conducted in more than half of the cases when the platelet count fluctuated from 10*10^9^/L to 30*10^9^/L. Immediate platelet transfusion was also indicated in the presence of significant clinical hemorrhage or if the platelet count was below 50*10^9^/L before cesarean delivery.

In a small pre-test, it was found that Rh(D)-positive patients with ITP without splenectomy tolerated intravenous anti-D well, and this effect was particularly significant in the middle and late pregnancy, where it was effective and safe for both mother and neonate at doses of 50–75 μg/kg (10, 11). In the ITP guide published by blood in 2019 do not include the above therapy in the recommended list of first-line treatments for ITP in pregnancy, but it is an option for non-pregnant adults and children with ITP, and it is not currently recommended in the Chinese guidelines. However, it is undeniable that many research teams are conducting IV anti-D-related clinical trials in China and abroad, and the research progress is worth expectation.

Most patients respond well to the first-line treatment options, and evidence-based medical data for second-line treatment options are scarce. Among the immunosuppressive agents, cyclosporine A and azathioprine are safe for application during pregnancy (12, 13). Thus, the application might be optionally considered by clinicians, but it might take several weeks to raise the platelet count and is not recommended in critical conditions or during the perioperative period. The application of Thermoplastic polyolefin(TPO) receptor agonists, such as romiplostim and eltrombopag, has a case report that has been reported in ITP in pregnancy (14–17), but the data are minimal and cannot yet be recommended as a standard second-line treatment option.

The main points of diagnosis and treatment by our obstetricians could be summarized as follows: for those with platelet counts no less than 50*10^9^/L during the whole pregnancy, or those with platelet counts at 30*10^9^/L or higher in middle and late pregnancy without clinical symptoms, treatment generally should not be conducted with emphasis on observation and monitoring. In clinical practice, clinicians should not only consider the level and trend of platelet count but also pay attention to whether the patient tends to hemorrhage. Treatment should be given when the platelet count is below 20*10^9^/L, even if there are no obvious clinical signs such as hemorrhage. Aggressive treatment is indicated during the perioperative period or in the presence of clinical hemorrhagic symptoms, even if the platelet count is higher than 30*10^9^/L. In severe cases, testing for immune-related antibodies should be performed during pregnancy, with attention to screening for systemic immune disorders such as SLE and APS and developing a targeted therapeutic plan.

### Management and Treatment Experience

The management program included managing patients with ITP before pregnancy and patients with thrombocytopenia after pregnancy.

The importance of preconception counseling should be emphasized in the management of patients with ITP before pregnancy. It has been shown that 52% of patients with ITP have a significant decrease in platelet counts during pregnancy. Meanwhile, as pregnancy progresses, 49% of pregnant women require treatment (18), and 54.2% of pregnant women with ITP have a platelet count falling to 80*10^9^/L (19). For patients with ITP, pre-pregnancy counseling regarding the possible disease progression due to pregnancy and the choice of treatment options during pregnancy is an essential step. Patients with a pre-pregnancy platelet count below 30*10^9^/L with progressive decline, spontaneous hemorrhagic symptoms and even difficult to control, or those who have failed to respond to platelet-raising therapy or have serious comorbidities should be advised to delay pregnancy.

In managing patients with thrombocytopenia occurring after pregnancy, the goal of preventive intervention is to reduce the risk of hemorrhage in the perinatal period and local anesthesia. In early, middle, and late pregnancy, multiple blood tests should be conducted in the asymptomatic patients, which should be recommended every 2–4 weeks and should be tested more frequently if thrombocytopenia is detected. As mentioned above, ITP that occurs after pregnancy tends to occur in early pregnancy, and the platelet count is usually below 100*10^9^/L. The management of this group is similar to the management of ITP in adults, and a multidisciplinary team with obstetricians, neonatologists, anesthesiologists, and hematologists is required. Patients with GT also have the possibility of a sudden and significant drop in platelet counts during late pregnancy. Thus it is recommended to check the routine blood test every two weeks, and most pregnant women without hemorrhagic manifestations and with platelet counts higher than 30*10^9^/L do not need any treatment before 36 weeks of pregnancy, and in principle, they can wait for a natural delivery. Treatment is required to maintain the platelets at safe levels only when a pregnant woman has hemorrhagic symptoms, has a platelet count below 30*10^9^/L, or needs to be raised to a range that ensures safe delivery. Perfect follow-up information should be established after delivery. For the patient with chronic or refractory ITP, it is recommended to consult a hospital specializing in hematology to prepare for the next pregnancy.

### Neonatal Alloimmune Thrombocytopenia

Neonatal alloimmune thrombocytopenia (NAIT) is caused by the IgG autoantibodies produced by the mother with ITP, which enter the fetal circulation through the maternal–fetal barrier, destroying fetal platelets and causing NAIT (20). Most newborns with NAIT present at birth with subcutaneous or mucosal bleeding thrombocytopenia within the first 72 h of life. The most serious consequence is intracranial hemorrhage, with a prevalence of approximately 0.1%, and intracranial hemorrhage and intrauterine death can occur as early as 14–16 weeks of gestation (21). The majority of neonatal hemorrhagic events, such as intracranial and gastrointestinal hemorrhages, are due to NAIT, it is the same in full-term newborns (22). There are also a significant number of mild to moderate cases (with neonatal platelet counts >50*10^9^/L) that do not manifest with skin petechiae or bruises and are easily neglected (21). In the present study, although there were cases with neonatal thrombocytopenia, it was unclear if the diagnosis could be fully compatible with NAIT, but as a supplement, the authors made a space to present the analysis.

During pregnancy, women with ITP may lead to severe thrombocytopenia (<20*10^9^/L) in approximately 4% of newborns at birth. In those with moderately reduced platelets (<50*10^9^/L), the percentage can reach 9% (23), and in mildly reduced cases (<150*10^9^/L), the percentage is approximately 25.2–28% (9, 24). In the present study, 28.2% (11/39) of the neonates in group A suffered a mild reduction in platelet counts after birth. Among them, five neonates (approximately 12.8%) had platelet counts below 50*10^9^/L, and only one neonate (approximately 2.6%) had a platelet count below 20*10^9^/L. Another study with statistical analysis of the cases with severe thrombocytopenia in pregnant women revealed that six newborns had platelet counts below 50*10^9^/L, accounting for 5.7% (25). So far, no clear correlation between maternal and neonatal platelet counts has been reported in the literature. In the present study, the platelet counts in newborns in groups A and B were evenly distributed, and the platelet counts in the mothers and neonates might be inconsistent. No prenatal diagnostic method can effectively predict the postnatal condition of the newborn, and maternal treatment with glucocorticoids or IVIG does not affect the platelet count in the newborn (9). Even if the mother responds well to platelet-raising regimens during pregnancy, there is no guarantee of a total postnatal gain in the outcome of the newborn (26). The occurrence of thrombocytopenia in siblings at birth is a strong predictor for NAIT. Maternal splenectomy has also been reported to increase the risk of NAIT (27, 28). An 11-year retrospective study also found that children with NAIT would also be in remission in the future (24). Anti-platelet antibodies could be gradually cleared from the body in the newborn after birth, and platelets could return to the normal range after two months (26) even though these infants still face significant challenges to survive the perinatal period. One study found high levels of IgA-type anti-platelet antibodies in the breast milk of mothers with ITP, and these antibodies might also be associated with neonatal thrombocytopenia (29).

### Limitations in the Present Study

There existed the following limitations in the present study. The retrospective study limited the completeness of patient information throughout pregnancy and follow-up information at specific gestational ages. The wide range of patients admitted to our hospital resulted in poor patient follow-up and failure to ensure compliance. The small sample size limited further grouping and analysis.

## Summary

In summary, the differential diagnosis of thrombocytopenia of unknown cause in pregnancy is complex, the impact on the short-term outcome of the mother and neonate is not fully understood, and the long-term prognosis still should be explored in numerous clinical studies. A high degree of vigilance should be paid to the presence of moderate to severe thrombocytopenia during pregnancy, and primary care physicians should do a better job of early recognition and referral to prevent a progressive decline in platelet counts. Close observation of platelet changes during pregnancy should be conducted, with outpatient follow-up in place to improve compliance and administration of glucocorticoids, immunoglobulins, and/or platelet transfusions when necessary. Weighing the advantages and disadvantages at delivery, choosing the appropriate mode of delivery, and extending the follow-up period might be essential to reduce complications during pregnancy and improve maternal and child outcomes.

## Data Availability

The raw data supporting the conclusions of this article will be made available by the authors, without undue reservation. The datasets of this article described in the manuscript, including all relevant raw data, will be freely available to any scientist wishing to use them for non-commercial purposes, without breaching participant confidentiality.
